# Efficient Production of sTNFRII-gAD Fusion Protein in Large Quantity by Use of the Modified CHO-S Cell Expression System

**DOI:** 10.1371/journal.pone.0111229

**Published:** 2014-10-23

**Authors:** Qinzhen Cai, Ai Zhao, Yuting Yin, Lisha Ma, Zhenzhen Jiao, Huilin Zhi, Shouhua Lai, Sha Cheng, Hongmei Yang, Yinxiang Lu, Katherine A. Siminovitch, Jimin Gao

**Affiliations:** 1 Zhejiang Provincial Key Laboratory for Technology & Application of Model Organisms, School of Laboratory Medicine & Life Sciences, Wenzhou Medical University, Wenzhou, Zhejiang, China; 2 Departments of Immunology and Molecular Genetics, University of Toronto, Mount Sinai Hospital, Samuel Lunenfeld Research Institute and Toronto General Research Institutes, Toronto, Ontario, Canada; King's College London, United Kingdom

## Abstract

TNFα is one of the initial and important mediators to activate downstream signaling pathways by binding to trimerized TNFα receptors (TNFR), and thus is an ideal drug target for cancer therapy. Taking advantage of intrinsic homotimerization of the globular domain of adiponectin (gAD), we have developed a novel TNFα antagonist, the trimerized fusion protein named sTNFRII-gAD. However, our previously-used CHO expression system yielded less than 10 mg/L of sTNFRII-gAD. To produce large quantities of sTNFRII-gAD efficiently, we used a modified CHO-S cell expression system, which is based on a pMH3 vector with non-coding GC-rich DNA fragments for high-level gene expression. We obtained stable clones that produced 75 mg/L of sTNFRII-gAD in the 96-well plate, adapted the clones to 40 ml suspension serum-free batch culture, then optimized the culturing conditions to scale up the fed-batch culture in a 3 L shake-flask and finally in a 5 L AP30 bioreactor. We achieved a final yield of 52 mg/L of sTNFRII-gAD. The trimerized sTNFRII-gAD exhibited the higher affinity to TNFα with a dissociation constant (Kd) of 5.63 nM than the dimerized sTNFRII-Fc with a Kd of 13.4 nM, and further displayed the higher TNFα-neutralizing activity than sTNFRII-Fc (*p*<0.05) in a L929 cytotoxicity assay. Therefore, the strategy employed in this study may provide an efficient avenue for large-scale production of other recombinant proteins by use of the modified CHO-S cell expression system.

## Introduction

As an important cytokine, TNFα plays a pivotal part in many pathophysiological processes. In tissues, TNFα at low concentration shows beneficial effects, such asaugmentation of host defense mechanisms against infections, but at high concentration leads to inflammation and organ injury so as to cause diseases such as rheumatoid arthritis (RA), Crohn’s disease (CD), and psoriasis, etc. Moreover, acute release of large amounts of TNFαduring sepsis may result in septic shock. Therefore, neutralization of TNFα has become an effective therapeutic strategy for these diseases. The TNFα antagonists approved by the US FDA for clinical use include bivalent sTNFRII-Fc (Etanercept) and two monoclonal anti-TNFα antibodies (Infliximab and Adalimumab) [1], [2]. TNFα exerts its effects by binding, as a trimer, to either TNFRI or TNFRII. sTNFRII, the extracellular portion of TNFRII, is a natural TNFα antagonist [3]. However, monomeric sTNFRII has a lower affinity to TNFα, and also has a relative short half-life in circulation, thus resulting in lower therapeutic effects. It was reported that sTNFRII-Fc fusion protein could neutralize TNFα 50 to 1,000 times as much as monomeric sTNFRII due to the dimerization through the Fc moiety [4]. Therefore, enhancing the interaction between TNFα and sTNFR has become one of the major approaches to develop novel TNFα antagonists.

Adiponectin (AD), a 30 kDa protein hormone consisting of a globular domain (gAD) and a collagenous domain, originates from adipose tissue and regulates numerous metabolic processes. The gAD is located at the carboxyl terminus and inclines to form a homotrimer. A collagenous domain within AD leads to spontaneous self-assemblage into various oligomeric isoforms, including hexamers and high-molecular-weight multimers. The contribution of varying isoforms of AD to specific physiological processes remains to be fully elucidated. Two membrane-spanning receptors for AD have been identified in various body tissues with differing distribution density. The major intracellular pathway activated by AD includes the phosphorylation of AMP-activated protein kinase, which is responsible for many of its metabolic regulatory, anti-inflammatory, vascular protective and anti-ischemic properties. Since its discovery in 1995 AD has garnered considerable attention for its role in diabetic and cardiovascular pathology. Clinical observations have demonstrated the association of hypoadiponectinemia in patients with obesity, cardiovascular disease and insulin resistance [5]–[9].

Utilizing the intrinsic trimerization property of gAD, we have developed a novel TNFα antagonist, the trimerized fusion protein named sTNFRII-gAD, which was composed of sTNFRII and gAD. We had shown that sTNFRII-gAD was superior to sTNFRII-Fc as a TNFα antagonist, highlighting the potential of sTNFRII-gAD for the treatment of excessive TNFα-associated diseases [10]. However, our previously reported sTNFRII-gAD expression system yielded less than 10 mg/L of sTNFRII-gAD. In an effort to produce large quantities of recombinant sTNFRII-gAD for further studies, here we reported the construction of a modified CHO-S expression system based on “GC-rich” vector pMH3 for high-level gene expression [11], and further developed a high density, full suspension serum-free fed-batch culture system for production of sTNFRII-gAD in large quantity with high yield.

## Materials and Methods

### Materials and Instruments

Restriction enzymes EcoRI, NotI, and T4 DNA ligase were purchased from Takara (Shiga, Japan). *E.coli* DH5α competent cells and pAAV2-sTNFRII-gAD vector were generated by our own laboratory. pMH3 vector, B001 serum-free basal medium, F001 feed medium, frustoconical-bottom shake bottles, and AP30 bioreactor were provided by Amprotein (Hangzhou, Zhejiang, China). DMEM/F12 medium and fetal bovine serum (FBS) were from Gibco (Grand Island, NY, USA). Salmon sperm DNA was from Invitrogen (Carlsbad, CA, USA). G418 was from MerckChina (Shanghai, China).

### Cell Lines and Culture Conditions

The Chinese hamster ovary cell line (CHO-S) was kindly provided by AmProtein (Hangzhou, Zhejiang, China). CHO-S cells were grown in DMEM/F12 medium containing 10% FBS. For sTNFRII-gAD fusion protein expression, B001 serum-free basal medium and F001 feed medium were used. L929 cell was from ATCC (Manassas, VA, USA).

### Plasmid Construction

The *sTNFRII-gAD* encoding gene was amplified by PCR using pAAV2 -sTNFRII-gAD as template and under the following conditions: an initial denaturization of 2 min at 94°C was followed by 30 cycles of 15 s at 94°C, 30 s at 55°C, and 75 s at 68°C. Then a final elongation was performed for 10 min at 72°C. The forward and reverse primers were as follow: 5′-ACG *GAA TTC* GCC ACC ATG GCC CCC GTG GCC GT-3′ and 5′-AAA GAG ATA *TGC GGC CGC* TTA TCA TCA GTT GGT GTC GTG GTA CAG C-3′ (the underlined nucleotides of the primers denote the EcoRI and NotI sites, respectively). The PCR product was digested with EcoRI and Not I and then ligated to the expression vector pMH3, which was previously digested with these two enzymes. The expression plasmid pMH3-sTNFRII-gAD was purified from *DH5α* and the sequence of the resulting expression plasmid pMH3-sTNFRII-gAD was confirmed by DNA sequencing (Shanghai Bioengineering).

### Acquirement of Stable sTNFRII-gAD-expressing Cell Clones with High Yield

The expression plasmid pMH3-sTNFRII-gAD was introduced into CHO-S cells by a gene pulser (Hercules, CA, Bio-rad). CHO-S cells were harvested by centrifugation (800 rpm, 3 min) and washed with PBS twice, then 5×10^6^ cells were gently resuspended in 200 µl PBS. 25 µg pMH3-sTNFRII-gAD plasmid combined with 10 µl salmon sperm DNA was thoroughly mixed with 200 µl CHO-S cell suspension, which then was transferred into a chilled gene pulser cuvette. After 1 min on ice, electric shock with 160 V, 15 ms, followed by 1 min on ice immediately, then electric shock once again, the cell suspension was transferred into two 10 cm culture dishes with DMEM/F12 containing 10% FBS. After 24 h, the medium was replaced with selection medium containing 1.5 mg/ml G418 and 10% FBS. After about two weeks, neomycin resistant CHO-S/pMH3-sTNFRII-gAD cells were obtained. Following the above selection method, we obtained stable high expression clones in CHO-S in only one round G418 selection. The 2nd or 3rd clone selection was performed in order to get pure cell population. Subsequently, we acclimated the highest expressing clones to suspension culture in serum-free medium B001, then scaled up by using preliminarily optimized fed-batch cultures.

### Production of sTNFRII-gAD

#### (i) Serum-free suspension batch culture

The hyper-expression cells were seeded at a concentration of 2×10^6^ cells/ml and cultured in frustoconical-bottom shake flasks with a working volume of 40 ml with serum-free medium B001 on a shaker with an agitation of 120 rpm at 37°C. On the first day of batch cultures, 8 g/L glucose was added. The cell growth, viability and sTNFRII-gAD expression (by dot blot) were evaluated daily. When the cell viability dropped to 60%, the batch cultures were terminated.

#### (ii) Serum-free suspension fed-batch culture

In order to avoid nutrient limitations in suspension batch culture, we first performed fed-batch cultures in 3 L frosto-conical-bottom shake flasks and then scaled up to 5 L AP30 bioreactor. 100 ml and 2 L cultures of 2×10^6^ cells/ml were inoculated to 3 L frosto-conical-bottom shake flasks and 5 L AP30 bioreactor with serum-free medium B001 on a shaker at 120 rpm and 55 rpm at 37°C, respectively. When the cell density reached to more than 4–6×10^6^ cells/ml, feed medium F001 was added semi-continuously (e.g. daily or twice daily) to keep the glucose concentration at 2 g/L; meanwhile, the temperature was gradually reduced to 34°C. The culture in AP30 bioreactor was controlled by an on-line computer: pH 7.00±0.1, dissolved oxygen (DO) of 55% air saturation, and agitation at 55 rpm. The samples were analyzed daily to determine the cell viability, density, and metabolites. They were also frozen for yield analysis later via dot blot. When the cell viability dropped to 60%, the fed-batch cultures were terminated and the supernatants were harvested, filtered with a 0.22 µm cellulose acetate filter, and stored at −20°C for future use.

### Purification of sTNFRII-gAD

The harvested supernatants were concentrated 20-fold at 4°C with Pellicon tangential flow ultrafiltration system (membrane with a 30,000 molecular weight cut-off, Millipore Corporation, USA). The concentrates were subsequently loaded onto a 15 ml Pharmacia Q Sepharose XL Fast Flow column (GE healthcare, USA) equilibrated with PBS (pH 7.4). The column was then washed with 5 bed volumes of PBS buffer. The bound proteins were subsequently eluted with 10 times the column volume of PBS buffer and a linear gradient (0–100%) of 1 M sodium chloride. The eluted proteins were concentrated two fold by PEG20000, filtered, and applied to a HiLoad 16/60 Superdex 200 preparative grade column (GE healthcare, USA) with PBS as the running buffer (pH 7.4). The flow rate was maintained at 1 ml/min and the eluants were monitored again by a UV detector at 280 nm. Furthermore, the molecular weight of the purified trimerized sTNFRII-gAD fusion protein was confirmed by ultracentrifuge analysis (Instrument of physics, Chinese academy of sciences).

### Western Blotting and Dot Blotting

For reducing/non-reducing SDS-PAGE analysis, samples were boiled in sample-buffer with or without DTT, respectively. The proteins were detected in 10% SDS–polyacrylamide gels by Coomassie brilliant blue staining or transferred onto a nitrocellulose membrane and was analyzed as described [12]. 5 µl of culture supernatants was also applied onto the nitrocellulose membrane for dot blot semi-quantitatively assay. Mouse anti-human TNFRII/adiponectin antibody (Abcam,San Francisco, USA) was used as the primary antibody at a concentration of 0.05 µg/ml in conjunction with a goat anti-mouse IgG-alkaline phosphatase-conjugated or a goat anti-mouse IgG-peroxidase-conjugated secondary antibody (Bio-Rad Laboratories) at a concentration of 1∶3,000. The alkaline phosphatase substrate was BCIP/NBT (Calbiochem Corporation). The peroxidase substrate was 3-amino-9-ethylcarbazole (ECL Western blotting detecting reagent, Amersham).

### Affinity Measurement for Binding of sTNFRII-gAD to TNFα

Surface plasmon resonance (SPR) measurements were performed using a Biacore 3000 instrument (Biacore International AB, Uppsala, Sweden). TNFα was immobilized on a CM5 sensor chip at concentrations of 50 µg/mL in 10 mM sodium acetate, pH 4.0, using N-hydrosuccinimide/1-ethyl-3(-3-dimethylaminopropyl) -carbodiimide hydrochloride (NHS/EDC) at a flow rate of 10 µl/min. Binding of the purified trimerized sTNFRII-gAD fusion protein and commercial sTNFRII-Fc to the immobilized TNFα was measured by using serial dilutions of sTNFRII-gAD and sTNFRII-Fc from 130 to 4.0625 nM at a flow rate of 30 µl/min, respectively. Then they were eluted by 10 mM sodium hydroxide at 30 µl/min for 30 s, followed by PBS-T (PBS+0.005% Tween) buffer stabilization for 3 min. The dissociation constant (Kd) was derived from a linear regression of steady state of 1/Response versus 1/*C* double reciprocal plots as well as by fitting of binding kinetics using a first-order Langmuir model.

### sTNFRII-gAD Bioassays

The biological activity of the sTNFRII-gAD fusion protein was assessed by a TNFα-induced L929 cytotoxicity assay. Briefly, L929 cells were plated in a 96-well plate (1.5×10^5^ cells/well) and were allowed to attach at 37°C in a 5% CO_2_ incubator. After 24 h,the medium were replaced with 100 µl of indicated concentrations of sTNFRII-gAD, which were diluted by four times concentration gradient with DMEM containing 10% FBS, 20 U TNFα and 20 µg/ml actinomycin D. After incubation for 24 h at 37°C in 5% CO_2_, 20 µl of 5 mg/ml 3-(4,5-dimethylthiazol-2-yl)- 2,5-diphenyl-tetrazolium bromide (MTT) solution was added to each well and incubated for four hours. The media was removed, and 100 µl of dimethylsulfoxide was added to each well to dissolve the formazan dye for 30 minutes at 37°C. Absorbance was measured at a test wavelength of 570 nm and a reference wavelength of 630 nm with a microplate reader. Commercial sTNFRII-Fc was used for comparison.

## Results

### Construction of the sTNFRII-gAD Expression Plasmid

**Figure 1:**
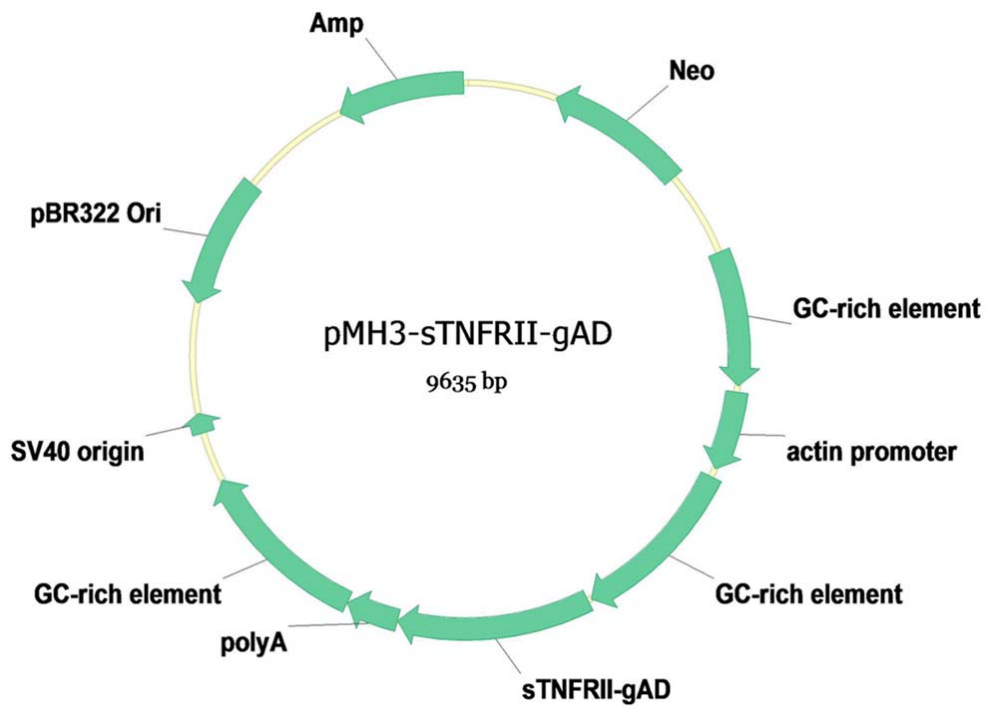
Schematic diagram of the recombinant plasmid pMH3-sTNFRII-gAD. GC-rich element: GC-rich non-coding DNA fragments; sTNFRII-gAD: soluble TNF receptor II and globular domain of adiponectin; actin promoter: chick beta-actin promoter; polyA: rabbit globulin polyA signal.

The CHO cell line was one of the most important systems for expression of foreign gene,but the expression level was low. Productivity of cell culture titer can be increased through the modulation of transcriptional activity via expression vector engineering by modulating the co-expression of product and selection marker genes, the stringency of the selection marker, the DNA regulatory elements carried on the vector, and targeting its integration site on the host cell genome [13]. To increase the protein yield, we chose pMH3 plasmid with GC-rich non-coding DNA fragments, which were crucial for chromatin openness [11]. The gene structure of pMH3-sTNFRII-gAD expression plasmid used for sTNFRII-gAD expression is shown in Fig. 1 and contains three GC-rich non-coding DNA fragments at the 5′ and 3′ flanking regions of *sTNFRII-gAD* gene and *actin* promoter.

### Selection of Stable sTNFRII-gAD-expressing Cell Clones with High Yield

**Figure 2:**
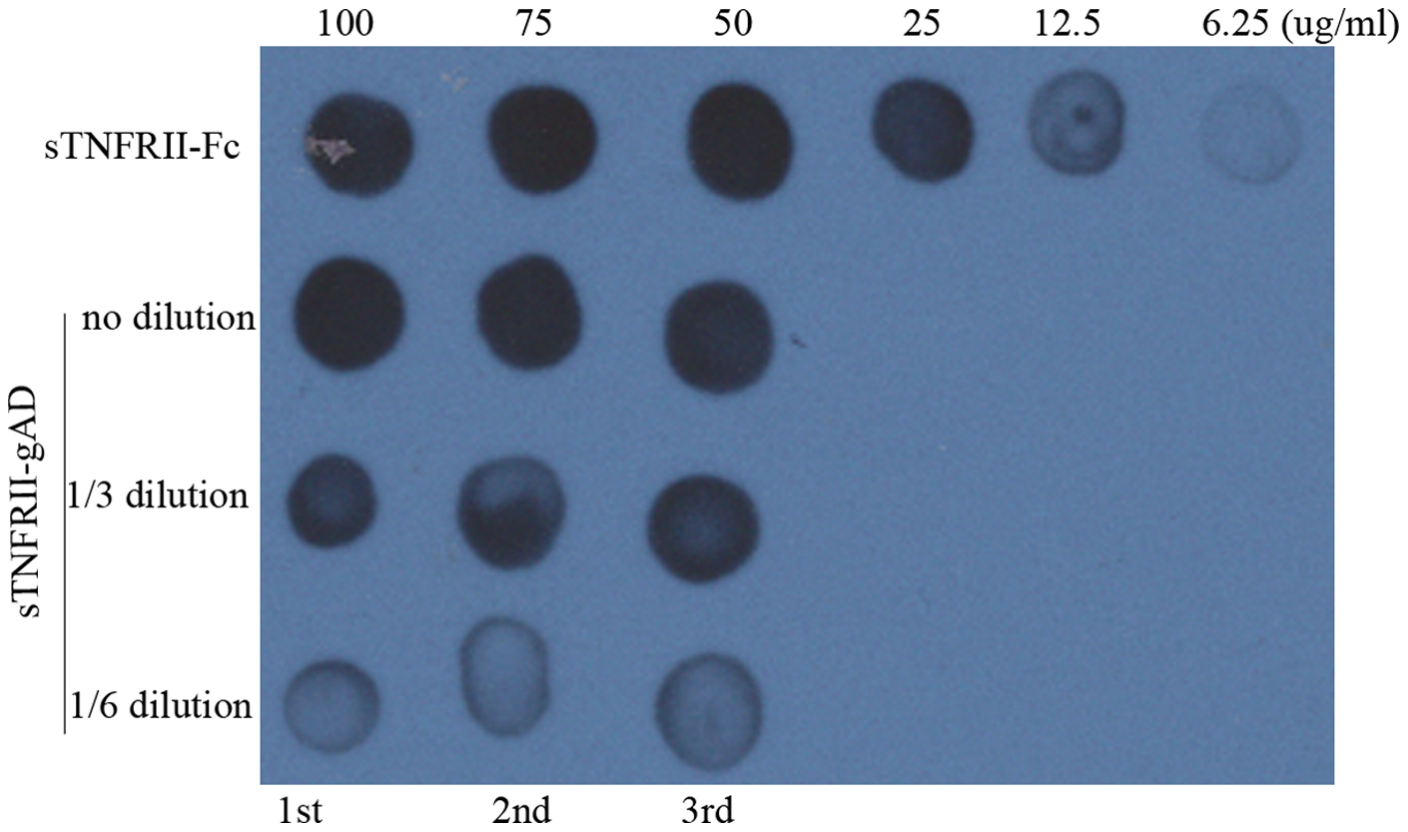
Dot blot analysis of sTNFRII-gAD protein in 96-well plates at 24 h with monoclonal antibody against TNFRII. Lane 1∶100, 75, 50, 25, 12.5 and 6.25 µg/ml of sTNFRII-Fc; Lane 2, 3, 4∶1/1, 1/3 and 1/6 dilutions of the supernatants from the 1st, 2nd and 3rd hyper-expression clones.

pMH3-sTNFRII-gAD plasmid was transfected into CHO-S cells via electroporation. The expression level of sTNFRII-gAD in 96-well plates reached up to 75 µg/ml after only one round of G418 selection in three weeks, as assessed by dot blotting analysis. Two rounds of clonal purification were undertaken subsequently, resulting in no dramatic change in expression level (Fig. 2).

**Figure 3:**
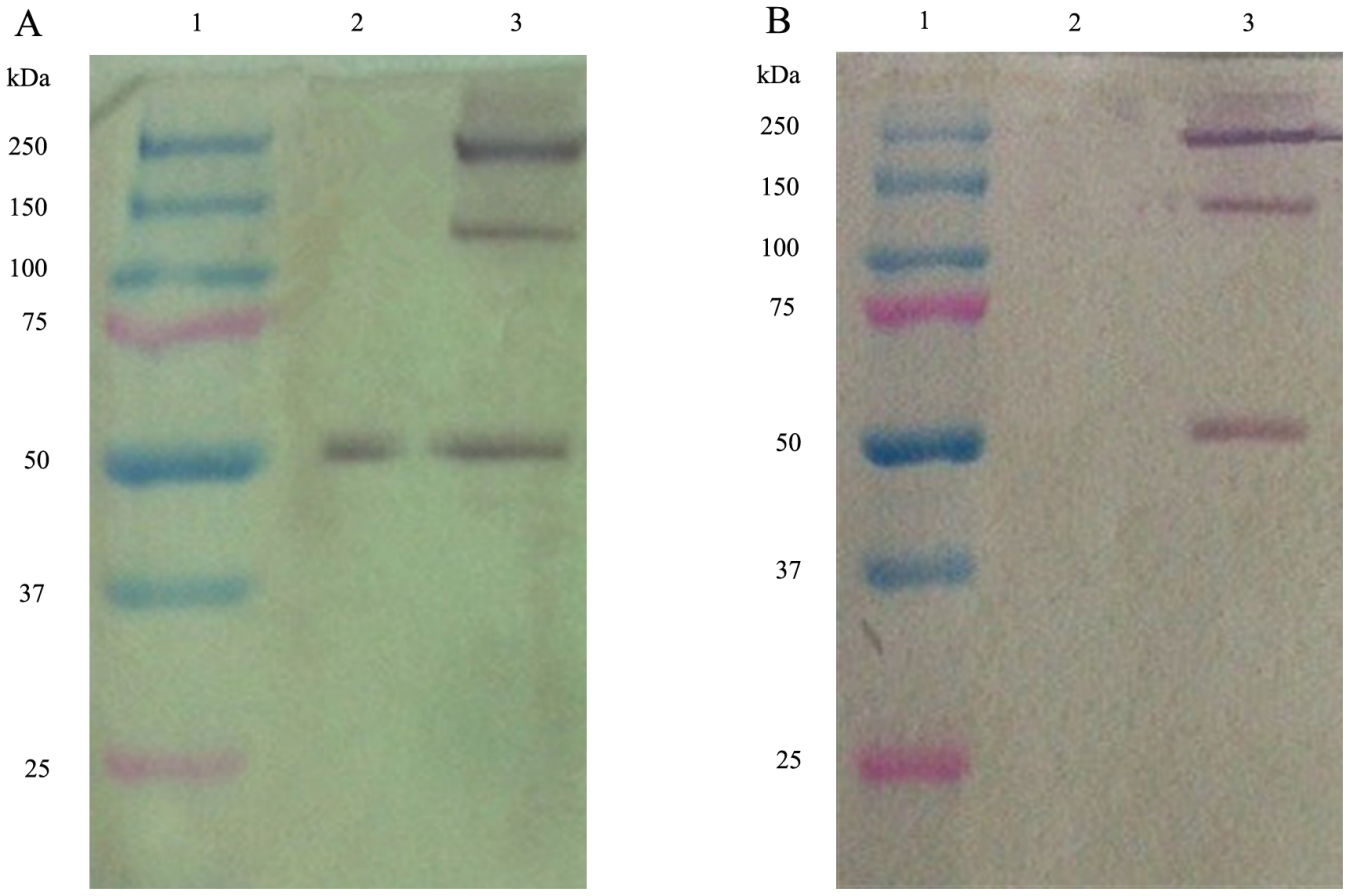
Western blot analysis of sTNFRII-gAD in culture supernatants using monoclonal antibody against adiponectin (A) and sTNFRII (B). Lane 1, molecular weight markers (kDa); Lane 2, reducing conditions; Lane 3, nonreducing conditions.

Furthermore, the supernatants from the selected hyper-expression clones were analyzed by western blot with monoclonal antibodies against sTNFRII or adiponectin. Three antibody-specific bands with approximate apparent molecular weights of 50, 150, 250 kDa were identified in non-reducing conditions, which represented monomer, trimer, and multimer form of sTNFRII-gAD, respectively (Fig. 3A, 3B). In reducing conditions, there existed one specific bands with approximate apparent molecular weights of 50 kDa, the band was recognized by anti-adiponectin (Fig. 3A), while not by anti-TNFRII (Fig. 3B). This may due to the fact that anti-TNFRII could only recognize spatial epitope of sTNFRII (not linear epitope), while anti-adiponectin could identify linear epitope of gAD [14].

### Production of sTNFRII-gAD Fusion Protein

**Figure 4:**
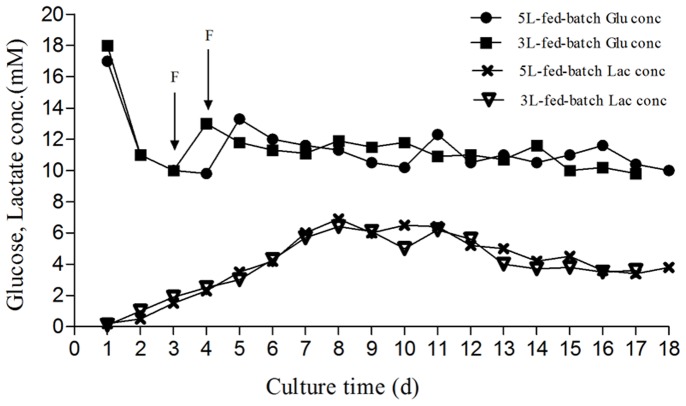
The metabolism of glucose and lactate of sTNFRII-gAD-expressing CHO-S cells in 3L and 5L fed-batch cultures in shaker bottles and AP30 bioreactor. (F: feed medium F001).

The hyper-expression clones were adapted to high density, serum-free suspension culture by use of chemically-defined medium that was capable of producing sTNFRII-gAD. The culturing time of suspension batch was much shorter than that of fed batch. The yields of sTNFRII-gAD in the fed batch were about 3–4 times more than that in suspension batches (Table 1). High density, high viability, and high yields in fed-batch cultures may be mainly due to the preliminarily optimized feeding strategy, such as high inoculation density of 2×10^6^ cells/ml, later time to feed as the cell density expanding to 6–8×10^6^ cells/ml, maintenance of lower glucose level of 2 g/L (11 mM), and decrease in temperature to 34°C at the production stage. We found that kept glucose level at 2 g/L resulted in low lactate concentration, which may prevent cell death (Fig. 4). We also found that when fed batch scaled up to 5 L in an AP30 bioreactor, cells still remained high density, high viability, and high yields, indicating that the performances achieved on the small scales (3 L) could be reliably expanded to the large scale.

**Table 1:** Comparison of the Growth and sTNFRII-gAD Fusion Protein Production of the Engineered Cells in Suspension Culture.

	40 ml batch	3 L fed-batch	5 L fed-batch
Culture time (d)	8.00	17.00	18.00
Max conc (10^6^ cells/ml)	6.00	9.20	8.90
Supernatants collected (L)	0.04	1.80	4.60
Protein concentration (mg/L)^a^	13.00	40.00	52.00
Protein purity (%)^b^	90.00		

a Estimated by BCA protein assay after anion exchange chromatography.

b Determined by densitometry of gel (data not shown).

### Purification of sTNFRII-gAD Fusion Protein

**Figure 5:**
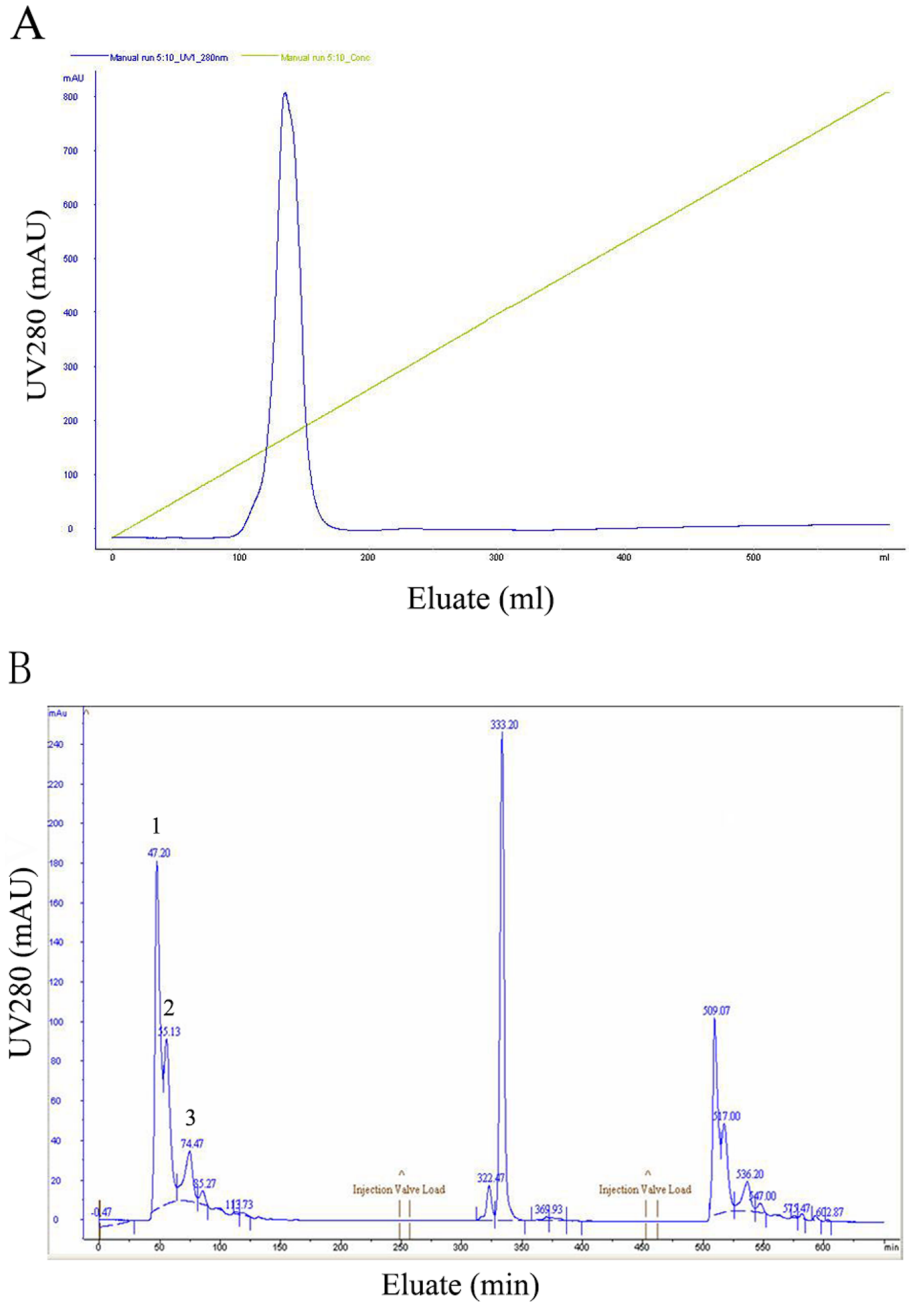
The first-step purification of sTNFRII-gAD by anion exchange chromatography (A). The second-step purification of sTNFRII-gAD by a size exclusion chromatography (B). Peak 1: multimeric sTNFRII-gAD; peak 2: trimeric sTNFRII-gAD; peak 3: monomeric sTNFRII-gAD.

**Figure 6:**
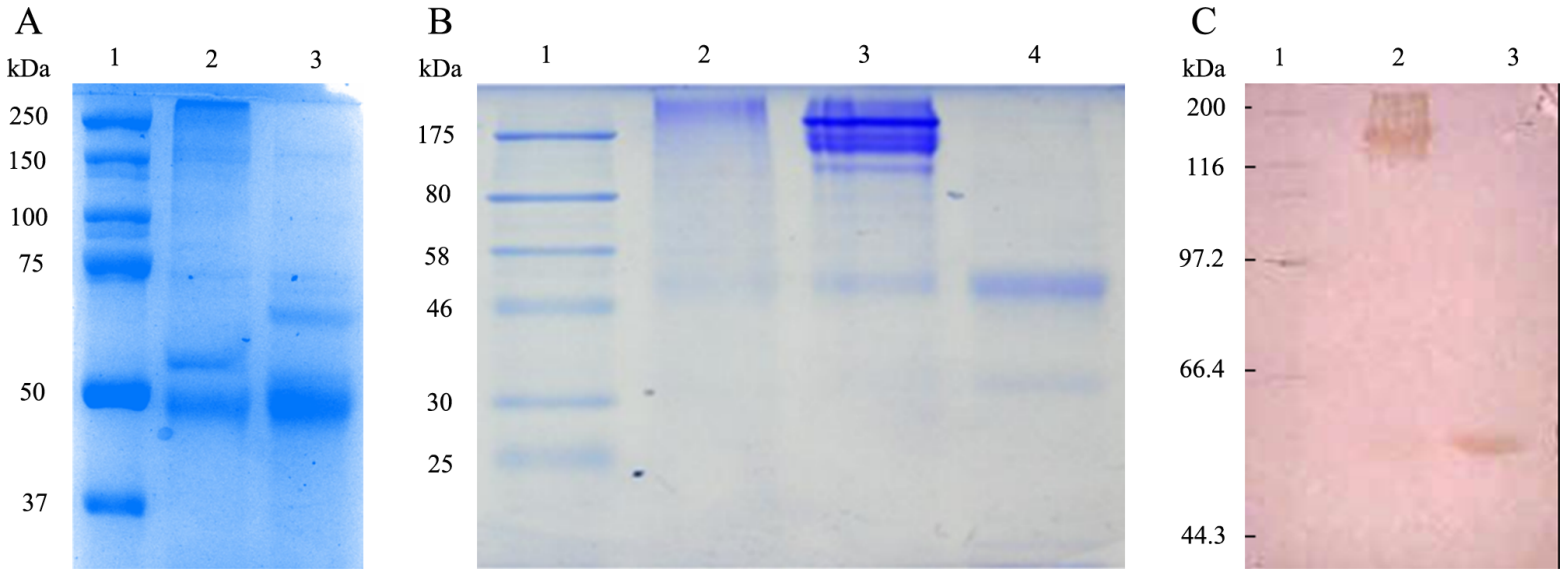
SDS-PAGE and western blot analyses of the purified sTNFRII-gAD. (A) SDS-PAGE analysis of sTNFRII-gAD purified by anion exchange chromatography. Lane 1:molecular weight markers (kDa); lanes 2, 3: The loaded protein samples were about 5.0 µg each loading in non-reducing and reducing conditions, respectively. (B) SDS-PAGE analysis of sTNFRII-gAD separated by HiLoad 16/60 Superdex 200 chromatography. Lane 1, protein molecular weight markers; Lane 2, multimeric sTNFRII-gAD (peak 1); Lane 3, trimeric sTNFRII-gAD (peak 2); Lane 4, monomeric sTNFRII-gAD (peak 3). The loaded protein samples were about 2.0, 5.0, and 3.0 µg, respectively. (C) Western blot analysis of purified trimeric sTNFRII-gAD under non-reducing/reducing conditions. Lane 1, molecular weight markers; Lanes 2 and 3 The loaded protein samples were about 5.0 µg and 3.0 µg in non-reducing and reducing conditions, respectively.

**Figure 7:**
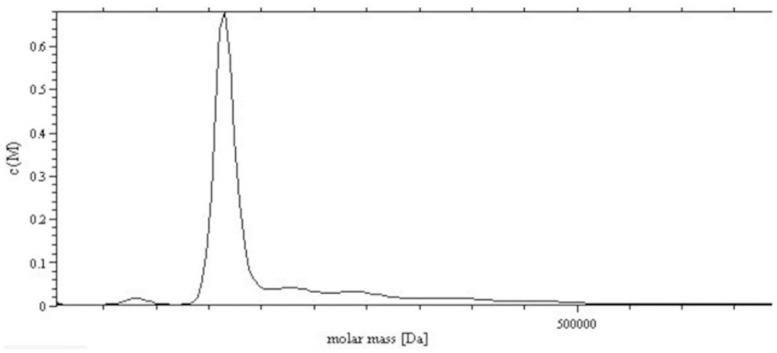
Ultracentrifuge analysis of the trimerized sTNFRII-gAD. It gave a molecular weight of 165 kDa.

The harvested supernatants were concentrated by 20-fold through a cascade TFF system before sTNFRII-gAD fusion protein purification. The concentrates were subsequently loaded onto an anion exchange column. The elution revealed one major protein peak (Fig. 5A). The eluted sTNFRII-gAD fusion protein showed three major bands on SDS-PAGE gel with approximate apparent molecular weights of 50, 150, 250 kDa, which represented monomer, trimer, and multimer forms, respectively (Fig. 6A). The eluted protein was further purified to homogeneity using a size exclusion column, giving rise to three major protein peaks (Fig. 5B). SDS-PAGE analysis revealed that the peaks 1, 2, 3 were multimers, trimers, and monomers respectively (Fig. 6B). The identity of the purified sTNFRII-gAD was further confirmed by western blot assay (Fig. 6C), as well as by N-terminal sequencing (L-P-A-Q-V). Furthermore, ultracentrifuge analysis of the trimerized sTNFRII-gAD indicated a molecular weight of 165 kDa (Fig. 7), consistent with the SDS-PAGE result (150 kDa).

### Binding Affinity of sTNFRII-gAD to TNFα

**Figure 8:**
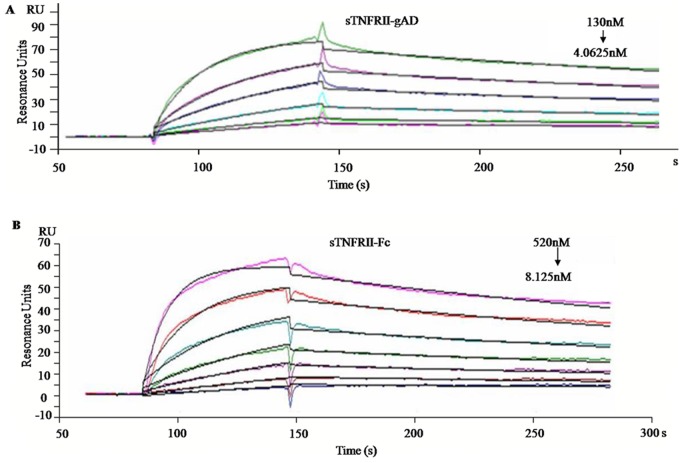
Biacore sensorgram of sTNFRII-gAD/sTNFRII-Fc binding to TNFα immobilized on a CM5 chip. Binding measurements were performed using serial dilutions of sTNFRII-gAD and sTNFRII-Fc. The concentrations of sTNFRII-gAD were 130, 65, 32.5, 16.25, 8.125, and 4.0625 nM (A), while the concentrations of sTNFRII-Fc were 520, 260, 130, 65, 32.5, 16.25, and 8.125 nM (B).

Direct binding of recombinant sTNFRII-gAD/sTNFRII-Fc to TNFα was measured through Biacore technology. By analysis of the sensorgram cruve, the apparent dissociation constant (Kd) of the purified trimered sTNFRII-gAD and a commercial sTNFRII-Fc for TNFα were 5.63 nM and 13.4 nM, respectively (Fig. 8), with on kinetic constants of 1.83e5 1/ms and 4.32e5 1/ms, and off kinetic constants of 2.45e-3 1/s and 2.43e-3 1/s for sTNFRII-gAD and sTNFRII-Fc, respectively.

### Biological Activity of sTNFRII-gAD Fusion Protein

**Figure 9:**
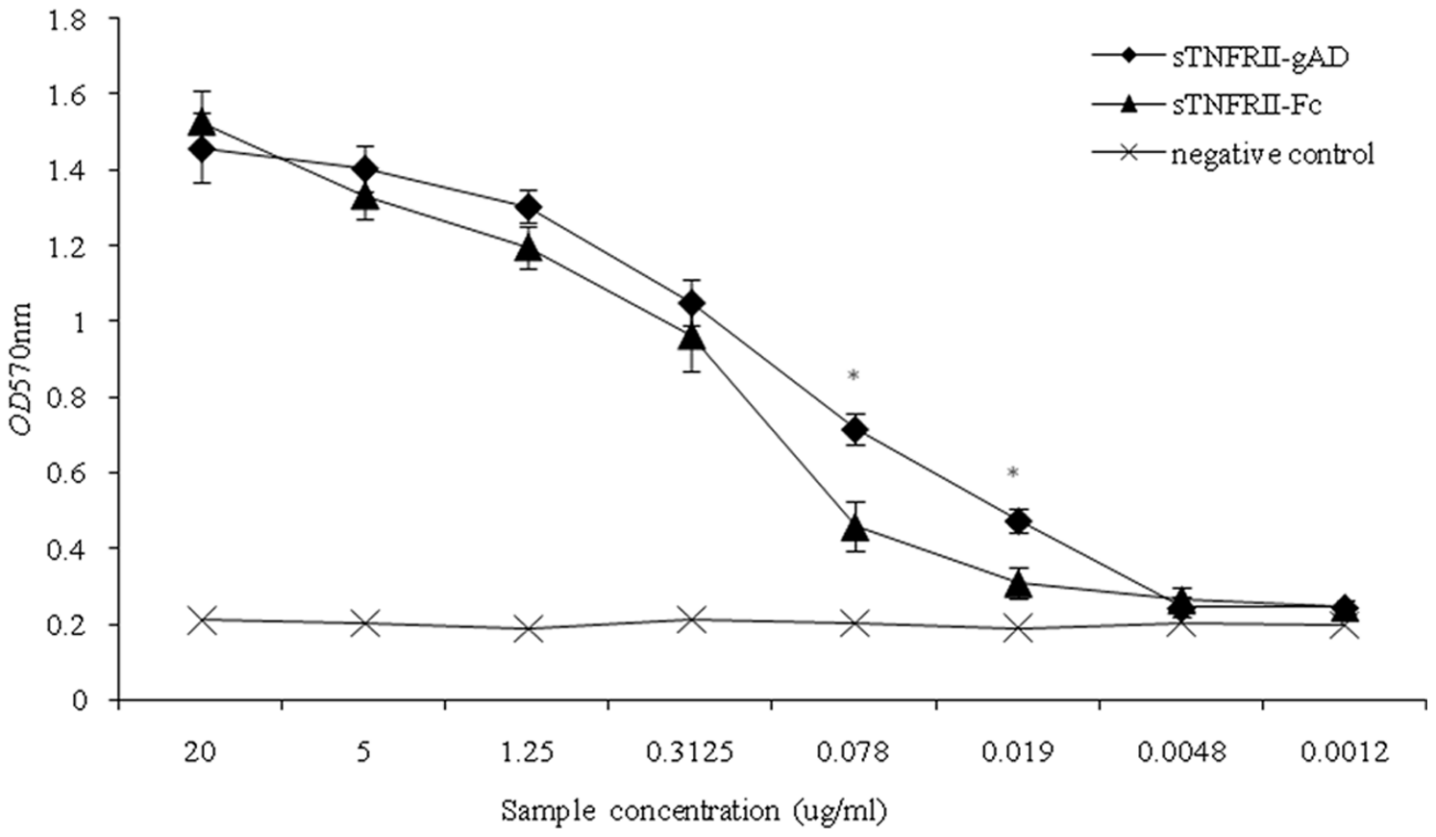
Biological activity of purified sTNFRII-gAD. An asterisksymbol (*) indicated a statistically significant difference (*p*<0.05) between sTNFRII-Fc and sTNFRII-gAD. All assays were performed in triplicate, with error bars representing the standard error of the mean of the samples.

Bioassays were conducted to measure the biological ability of the purified recombinant sTNFRII-gAD fusion protein to antagonize TNFα-induced L929 cytotoxicity, and compare its activity with sTNFRII-Fc. The dose-response curves showed that both sTNFRII-gAD and sTNFRII-Fc neutralized TNFα effectively in a dose-dependent manner. Neutralizing activity of sTNFRII-gAD was significantly higher than that of sTNFRII-Fc (*p*<0.05) (Fig. 9), which is consistent with our previous observation showing that sTNFRII-gAD was able to attenuate D-galactosamine/LPS-induced acute liver injury resulted from excessive TNFα more efficaciously than sTNFRII-Fc [10].

## Discussion

A novel TNFα antagonist, sTNFRII-gAD, was first successfully expressed in a mammalian expression system in our lab, although with low yields [15]. Subsequently, we modified the coding sequence of the sTNFRII-gAD gene in order to increase the expression level without altering the amino acid sequence, followed by using a dihydrofolate reductase gene for gene amplification to increase protein express in CHO/*dhfr* ^−^ cells. However, the yield reached to only 8 mg/L in bioreactors [15].

In this report, high level expression of recombinant sTNFRII-gAD fusion protein was achieved by use of a “GC-rich” expression vector. An alternative non-coding GC-rich DNA fragment was proposed to be a novel ubiquitous chromatin opening elements (UCOE), since flanking the gene of interest with the GC-rich fragment augmented recombinant protein expression [11]. UCOE was reported to prevent gene silencing consistently and resulted in stable and high expression of the gene of interest regardless of the chromosomal integration site [16]. Use of the pMH3-sTNFRII-gAD construct resulted in 75 µg/ml of sTNFRII-gAD in a 96-well plate after only one round of G418 selection. More importantly, the expression levels in scale-up cultures were relatively stable, with a final yield of 52 mg/L (Table 1). This represents a 5 to 6-fold improvement in sTNFRII-gAD production as compared with our previous expression system, the most commonly used CHO/DHFR expression system. The system suffered from requiring a high throughput screening to improve the yield by increasing the gene copy, loss of the gene of interest during long-term culture, toxicity, high cost, and complicated downstream purification associated with MTX [17]. The approach reported here avoids these shortcomings, thus will greatly shorten the research and development cycle of biopharmaceuticals and reduce labor and cost.

Nowadays, serum-free suspension culture technology has become the mainstream in biopharmaceutical industry. Suspension fed-batch culture is frequently employed due to the fact that it is easy to operate, scale up, control and concentrate the end-products. Compared to batch culture, fed-batch culture could increase viable cell density, prolong culture time, and allow product accumulation to a higher concentration. After selecting the final stable hyper-expression clones in 96-well plates, the cells need to adapt to suspension culture in serum-free medium B001 for large-scale culture. Moreover, well-controlled cell cultures at small scale could provide crucial information to assess and resolve unexpected problems in large-scale system [18]. To maintain high cell viability and high yield, we selected stable clones for 40 ml batch culture, then optimized the culturing conditions and scaled up to fed-batch cultures in 3 L shake flasks and 5 L bioreactor sequentially. The appropriate feeding strategy was of great importance, such as the starting time of feeding and feeding volume and temperature. Though there would be no nutrition limits, premature feeding could lead to excessive nutrients in the culture system, which increased the osmotic pressure and might inhibit cell growth and result in lower cell density and viability in late period of the culture. The determination of a key substrate, e.g. glucose, would be ideal for feeding control. Previous studies indicated that the by-products, such as lactate, could decrease by maintaining low glucose concentrations through frequent or continuous feeding [19]. Due to metabolic shift, adapting cells to a low-glucose environment produced less lactate and improved protein production [20]–[22]. Additionally, lowering temperature is a effective method to control cell proliferation [23]. Several studies showed that the temperature shift from 37°C to 30–35°C at 48 hours post inoculation could retain cells in G1 phase longer, and therefore delayed the onset of apoptosis and enhanced cell specific production [24]–[29]. Finally, the yields of sTNFRII-gAD fusion protein increased 3-4-fold in fed batch as compared with batch culture, close to that in 96-well plates.

In conclusion, we constructed a modified CHO-S expression system for sTNFRII-gAD fusion protein on basis of “GC-rich” vector pMH3 for high-level gene expression, and further developed a high density, full suspension serum-free fed-batch culture system for production of sTNFRII-gAD in large quantity with high yield. Thus, the strategy employed in this study may provide an efficient avenue for large-scale production of other recombinant proteins by use of the modified CHO-S cell expression system.

## References

[1] Landry Y, Gies JP (2008) Drugs and their molecular targets: an updated overview. Fundamental & Clinical Pharmacology 22: 1–18. 10.1111/j.1472-8206.2007.00548.x 18251718

[2] Toussirot É, Wendling D (2004) The use of αTNF-α blocking agents in rheumatoid arthritis: an overview. Expert Opinion on Pharmacotherapy. 5: 581–594. 10.1517/14656566.5.3.581 15013927

[3] Fernandez-Botran R (2000) Soluble cytokine receptors: novel immunotherapeutic agents. Expert Opinion on Investigational Drugs 9: 497–514. 11060691 10.1517/13543784.9.3.497

[4] Mohler KM, Torrance DS, Smith CA, Goodwin RG, Stremler KE, et al (1993) Soluble tumor necrosis factor (TNF) receptors are effective therapeutic agents in lethal endotoxemia and function simultaneously as both TNF carriers and TNF antagonists. The Journal of Immunology 151: 1548–1561. 8393046

[5] Combs TP, Wagner JA, Berger J, Doebber T (2002) Wang, (2002) et al Induction of Adipocyte Complement-Related Protein of 30 Kilodaltons by PPARγ Agonists: A Potential Mechanism of Insulin Sensitization. Endocrinology 143: 998–1007. 11861525 10.1210/endo.143.3.8662

[6] Maeda K, Okubo K, Shimomura I, Funahashi T, Matsuzawa Y, et al (1996) cDNA Cloning and Expression of a Novel Adipose Specific Collagen-like Factor, apM1 (AdiposeMost Abundant Gene Transcript 1). Biochemical and Biophysical Research Communications 221: 286–289. 8619847 10.1006/bbrc.1996.0587

[7] Hu E, Liang P, Spiegelman BM (1996) AdipoQ Is a Novel Adipose-specific Gene Dysregulated in Obesity. Journal of Biological Chemistry 271: 10697–10703. 8631877 10.1074/jbc.271.18.10697

[8] Heiker John T, Kosel D, Beck-Sickinger AG (2010) Molecular mechanisms of signal transduction via adiponectin and adiponectin receptors. Biological Chemistry. pp. 1005. 10.1515/BC.2010.104 20536390

[9] Scherer PE, Williams S, Fogliano M, Baldini G, Lodish HF (1995) A Novel Serum Protein Similar to C1q, Produced Exclusively in Adipocytes. Journal of Biological Chemistry 270: 26746–26749. 7592907 10.1074/jbc.270.45.26746

[10] Luo M, Liu D, Zhang L, Huang S, Yang W, et al (2012) Protective effects of a novel trimerized sTNFRII on acute liver injury. International Immunopharmacology 13: 88–92. 22465962 10.1016/j.intimp.2012.03.013

[11] Jia Q, Wu H, Zhou X, Gao J, Zhao W, et al (2010) A “GC-rich” method for mammalian gene expression: A dominant role of non-coding DNA GC content in regulation of mammalian gene expression. Science China Life Sciences 53: 94–100. 20596960 10.1007/s11427-010-0003-x

[12] Sambrook J, Fritsch EF, Maniatis T (1989 ) Molecular Cloning: a Laboratory Manual, second ed. New York: Cold Spring harbor laboratory Press.

[13] Lai T, Yang Y, Ng S (2013) Advances in Mammalian Cell Line Development Technologies for Recombinant Protein Production. Pharmaceuticals 6: 579–603. 24276168 10.3390/ph6050579 PMC3817724

[14] Chen S, He Q, Dong X, Wu X, Gao J (2010) [Eukaryotic expression and bioactivity determination of the fusion protein sTNFRII-gAD consisting of soluble tumor necrosis factor receptor II and globular domain of adiponectin]. Sheng wu gong cheng xue bao = Chinese journal of biotechnology 26: 207–215. 20432940

[15] Huang S, Yin Y, Xiong C, Wang C, Lü J, et al (2013) [Comparison of two types of cell cultures for preparation of sTNFRII-gAD fusion protein]. Sheng wu gong cheng xue bao = Chinese journal of biotechnology 29: 115–118. 23631125

[16] Allen ML, Antoniou M (2007) Correlation of DNA methylation with histone modifications across the HNRPA2B1-CBX3 ubiquitously-acting chromatin open element (UCOE). Epigenetics 2: 227–236. 18032920 10.4161/epi.2.4.5231

[17] Kim NS, Kim SJ, Lee GM (1998) Clonal variability within dihydrofolate reductase-mediated gene amplified Chinese hamster ovary cells: Stability in the absence of selective pressure. Biotechnol Bioeng 60: 679–688. 10099478

[18] Li F, Hashimura Y, Pendleton R, Harms J, Collins E, et al (2006) A Systematic Approach for Scale-Down Model Development and Characterization of Commercial Cell Culture Processes. Biotechnology Progress 22: 696–703. 16739951 10.1021/bp0504041

[19] Xie L, Wang DIC (1996) High cell density and high monoclonal antibody production through medium design and rational control in a bioreactor. Biotechnol Bioeng 51: 725–729. 18629840 10.1002/(SICI)1097-0290(19960920)51:6<725::AID-BIT12>3.0.CO;2-C

[20] Gambhir A, Europa AF, Hu W-S (1999) Alteration of cellular metabolism by consecutive fed-batch cultures of mammalian cells. Journal of Bioscience and Bioengineering 87: 805–810. 16232558 10.1016/s1389-1723(99)80157-1

[21] Wlaschin K, Hu W-S (2006) Fedbatch Culture and Dynamic Nutrient Feeding. In: Hu W-S, editor. Cell Culture Engineering: Springer Berlin Heidelberg. pp. 43–74. 10.1007/10_015 16989257

[22] Maranga L, Goochee CF (2006) Metabolism of PER.C6TM cells cultivated under fed-batch conditions at low glucose and glutamine levels. Biotechnol Bioeng 94: 139–150. 16523524 10.1002/bit.20890

[23] Reuveny S, Kim YJ, Kemp CW, Shiloach J (1993) Effect of temperature and oxygen on cell growth and recombinant protein production in insect cell cultures. Appl Microbiol Biotechnol 38: 619–623. 7763472 10.1007/BF00182800

[24] Furukawa K, Ohsuye K (1999) Enhancement of productivity of recombinant α-amidating enzyme by low temperature culture. Cytotechnology 31: 85–94. 19003128 10.1023/A:1008059803038 PMC3449780

[25] Moore A, Mercer J, Dutina G, Donahue C, Bauer K, et al (1997) Effects of temperature shift on cell cycle, apoptosis and nucleotide pools in CHO cell batch cultues. Cytotechnology 23: 47–54. 22358520 10.1023/A:1007919921991 PMC3449885

[26] Fox SR, Tan HK, Tan MC, Wong SCNC, Yap MGS, et al (2005) A detailed understanding of the enhanced hypothermic productivity of interferon-γ by Chinese-hamster ovary cells. Biotechnology and Applied Biochemistry 41: 255–264. 15504103 10.1042/BA20040066

[27] Yoon SK, Song JY, Lee GM (2003) Effect of low culture temperature on specific productivity, transcription level, and heterogeneity of erythropoietin in Chinese hamster ovary cells. Biotechnol Bioeng 82: 289–298. 12599255 10.1002/bit.10566

[28] Yoon SK, Hwang SO, Lee GM (2004) Enhancing Effect of Low Culture Temperature on Specific Antibody Productivity of Recombinant Chinese Hamster Ovary Cells: Clonal Variation. Biotechnology Progress 20: 1683–1688. 15575699 10.1021/bp049847f

[29] Bollati-Fogolín M, Forno G, Nimtz M, Conradt HS, Etcheverrigaray M, et al (2005) Temperature Reduction in Cultures of hGM-CSF-expressing CHO Cells: Effect on Productivity and Product Quality. Biotechnology Progress 21: 17–21. 15903236 10.1021/bp049825t

